# Synthetic genome shuffling of poxviruses in yeast generates novel poxvirus candidates with potential oncolytic properties

**DOI:** 10.3389/fbioe.2026.1943548

**Published:** 2026-10-14

**Authors:** Aimeric Agaoua, Camille Rey, Julie Hortelano, Anne-Isabelle Moro, Benoit Grellier, Philippe Erbs

**Affiliations:** Transgene, Illkirch-Graffenstaden, France

**Keywords:** oncolytic, poxvirus, shuffling, synthetic, tar, viruses

## Abstract

Oncolytic viruses represent a promising class of biotherapeutics, yet their clinical performance is often constrained by limited cytolytic potency and insufficient immune activation. To address these limitations, we developed a synthetic biology framework that applies genome-scale recombination in yeast to generate chimeric poxviruses derived from multiple non-human-pathogenic poxvirus lineages. We first established the near-complete assembly of a vaccinia virus genome within yeast and demonstrated its ability to yield a functional virus in human cancer cells, providing a modular foundation for genome engineering. Building on this platform, we combined genomic inputs from different poxvirus species to create a diverse library of chimeric constructs, a subset of which could be recovered as infectious particles. Phenotypic characterization revealed substantial variation among chimeras, including distinct plaque morphologies, spreading behaviors, and activity across human cancer cell lines, indicating the emergence of functional diversity rather than incremental modification. Whole-genome sequencing confirmed that individual chimeras integrated genetic segments from several parental species, validating the broad recombination capacity of the yeast-based system. Collectively, these results establish a conceptual advance in synthetic virology. Yeast-mediated genome shuffling enables the rapid generation of functionally diverse chimeric poxviruses from multispecies inputs, providing a flexible and scalable platform for the identification and development of future oncolytic poxvirus candidates. While the chimeric viruses described here do not yet contain the attenuating mutations required for tumor-selective replication, they provide a foundation for the engineering of next-generation oncolytic biotherapeutics.

## 1 Introduction

Oncolytic viruses (OVs) are natural or genetically modified organisms capable of selectively infecting and lysing tumor cells while sparing normal tissues. In addition to their direct cytolytic effects, OVs can stimulate antitumor immune responses, further contributing to their therapeutic potential (Santos Apolonio et al., 2021). Compared to conventional cancer therapies, OVs offer the promise of reduced toxicity and enhanced efficacy, positioning them as attractive agents in cancer treatment (Vähä-Koskela et al., 2007; Russell et al., 2012). A diverse array of viruses has been explored for oncolytic applications, including adenoviruses, poxviruses, herpes simplex viruses, coxsackieviruses, polioviruses, measles virus, and Newcastle disease virus. Several of them have reached the clinical trial stage. (Heise and Kirn, 2000; Fábián et al., 2007; Dobrikova et al., 2008; Dingli et al., 2014).

Although numerous OVs have entered clinical development, regulatory approval remains limited to only a few agents, most notably talimogene laherparepvec (T-VEC) for melanoma and, more recently, teserpaturev (G47Δ) for malignant glioma. Despite these important milestones, the clinical impact of OVs has so far been more modest than anticipated, and several biological and translational barriers continue to limit their widespread implementation. These include insufficient intratumoral spread, antiviral immune clearance, and inadequate induction of durable systemic antitumor immunity (Macedo et al., 2020; Appleton et al., 2025). Collectively, these challenges highlight the need for innovative strategies to develop more potent and effective oncolytic virotherapies.

Directed evolution, based on iterative viral passaging under selective pressure in cancer cells, has been used to improve infectivity, spread, safety, and tumor specificity (Garijo et al., 2014; Waqqar et al., 2023). Nevertheless, the resulting enhancements have often remained modest and are generally restricted to a limited set of selectable phenotypic traits (Zainutdinov et al., 2019). Moreover, such adaptive strategies are generally more effective with RNA viruses than with DNA viruses, due to their intrinsically higher mutation rates.

An alternative and promising approach is the generation of chimeric viruses through recombination between distinct viral strains or variants, enabling the combination of advantageous traits and potentially leading to more substantial improvements in oncolytic efficacy (Kuhn et al., 2008; El-Andaloussi et al., 2012; Shmulevitz et al., 2012; Ricordel et al., 2018a). The Poxviridae family, comprising 18 genera and 46 known species capable of infecting vertebrates, includes several viruses with inherent oncolytic properties (Ricordel et al., 2018b). This diversity makes poxviruses excellent candidates for genome shuffling to create novel chimeric viruses. Leveraging the genetic diversity within this family, new viral species with enhanced oncolytic properties can be engineered. Notably, three chimeric poxviruses: deVV5, CF17, and CF33, have been developed for cancer therapy, demonstrating improved oncolytic activity compared to their parental strains (O’Leary et al., 2018; Ricordel et al., 2018a; Hammad et al., 2020). These chimeric viruses were generated via homologous recombination during co-infection of human cancer cell lines with multiple wild-type poxviruses.

However, the combination of homologous recombination and selection in cell lines typically yields a limited number of chimeric viruses, primarily selected for replication efficiency. Other important oncolytic properties, such as extracellular enveloped virus (EEV) production or syncytia formation, are not adequately represented (Law et al., 2006; Burton et al., 2019). Transformation-associated recombination (TAR) is a powerful cloning technique that exploits the homologous recombination machinery of yeast to assemble large DNA sequences, enabling the integration of up to 500 kb from multiple fragments in a single step (Gibson et al., 2008; Kouprina and Larionov, 2008).

TAR has been successfully employed to generate reverse genetic systems for both DNA and RNA viruses, including, among others, potyviruses (11 kb), adenoviruses (32 kb), herpes simplex virus (152 kb), and human cytomegalovirus (235 kb) (Ketner et al., 1994; Desbiez et al., 2012; Brown et al., 2017; Oldfield et al., 2017; Vashee et al., 2017; Thi Nhu Thao et al., 2020; Labroussaa et al., 2021; Knickmann et al., 2024; Fuchs et al., 2025; Rey et al., 2025). These cloned genomes can subsequently be used to reconstitute infectious viruses in appropriate host cells. TAR has also recently been used for partial poxviruses genomes (Chen et al., 2025; Gao et al., 2025).

Here, we establish a poxvirus genome-shuffling platform grounded in TAR that generates chimeric genomes independently of selection constraints imposed by cell culture. We first assembled a near-complete vaccinia virus genome in yeast and validated its capacity to yield a functional virus in human cancer cells, anchoring a modular workflow from design to functional readout. We then used this platform to combine genomic inputs from multiple parental poxvirus strains that are non-pathogenic to humans in *Saccharomyces cerevisiae*, creating a set of chimeric constructs that, upon recovery, exhibited broad phenotypic and genotypic diversity, including varied plaque morphologies, spreading dynamics, and activity across human tumor cell lines, consistent with reshuffling of traits beyond incremental modification. Whole-genome sequencing confirmed the incorporation of segments from multiple parental species within individual chimeras, demonstrating extensive recombination and modular trait integration enabled by the yeast-based system. To our knowledge, this constitutes the first demonstration that TAR cloning can be used to generate chimeric poxvirus genomes, advancing synthetic virology from *ad hoc* co-infection recombination toward a scalable, platform-driven exploration of the viral design space for next-generation oncolytic biotherapeutics.

## 2 Materials and methods

### 2.1 Yeast strain

*Saccharomyces cerevisiae* VL6-48 (ATCC MYA-3666™, genotype: MATa his3-Δ200 trp1-Δ1 ura3-52 ade2-101 lys2 ψ^+^ cir°) was cultured in Yeast Peptone Dextrose Adenine (YPDA) medium containing 1% yeast extract, 2% peptone and 2% glucose, supplemented with 40 mg.L^-1^ adenine, at 30 °C with agitation. Transformed VL6-48 strains were selected and maintained in minimal synthetic defined (SD, Takara Bio, Japan) bases with amino acid lacking tryptophan (DO Supplement -Trp, Takara Bio, Japan) abbreviated SD-TRP. For solid media, 2% Bacto™ Agar (Dutscher, France) was added.

### 2.2 Bacterial strain

*Escherichia coli* EPI300 (LGC Biosearch Technologies, United States) was used for DNA cloning and grown in LB broth (Sigma-Aldrich, United States), with or without agar, supplemented when required with chloramphenicol at 34 μg.mL^-1^ and kanamycin at 50 μg.mL^-1^ (Sigma-Aldrich, United States).

### 2.3 Cell lines

Human tumor cell lines A549 (ATCC CCL-185), HeLa (ATCC CCL-2), MIA-PaCa-2 (ATCC CRL-1420), HCT116 (ATCC CCL-247), and the African green monkey kidney cell line Vero (ATCC CCL-81) were maintained at 37 °C in a humidified atmosphere of 5% CO_2_ in Dulbecco’s Modified Eagle Medium (DMEM, Thermo Fisher Scientific, United States) or McCoy’s 5 A medium (LGC Standards, United Kingdom), supplemented with 10% fetal bovine serum (FBS, Thermo Fisher Scientific, United States) and 100 μg.mL^-1^ gentamycin (Sigma-Aldrich, United States).

### 2.4 Viruses

Wild-type Vaccinia virus strain Copenhagen (VACV) was obtained from Institut Mérieux (France), while wild-type Cowpox virus strain Brighton (CPXV; ATCC VR-302) and wild-type Rabbitpox virus strain Utrecht (RPXV; ATCC VR-1591) were obtained from ATCC (United States). VACV, CPXV and RPXV stocks were propagated in HeLa cells using standard procedures. Modified Vaccinia virus Ankara (MVA) (Spehner et al., 2000) was amplified in primary chicken embryo fibroblasts (CEF) as previously described (Erbs et al., 2008).

### 2.5 Plasmid construction

The pYCC plasmid was constructed using the pCC1-FOS copy-control plasmid (LGC Biosearch Technologies, United States) as template. The yeast replication region *CEN6-ARS4* and the autotrophic selection gene TRP1 were amplified as a single DNA fragment from the pRS314 plasmid (Sikorski and Hieter, 1989) by PCR using Primestar DNA polymerase (Takara Bio, Japan). The PCR product was inserted into pCC-FOS1 by InFusion® cloning (Takara Bio, Japan) at the AfeI restriction site (Hou et al., 2016). pCC-FOS1 was digested with AfeI (New England Biolabs, United States) according to the manufacturer’s instructions. Linker sequences 5′SU-L3′ were designed using Geneious software (see Supplementary Table 1) and synthesized by GeneArt (Thermo Fisher Scientific, United States). The pYCC3 plasmid was generated by InFusion® cloning of EcoRI-HF–digested pYCC (New England Biolabs, United States) with linker 5′SU-L3′ following the manufacturer’s protocol, and the resulting products were chemically transformed into EPI300. Correct insertion was verified by Sanger sequencing (Eurofins Genomics, Germany).

### 2.6 Viral genome extraction

Viral DNA was extracted from 2 mL of purified virus using the following procedure: incubation for 2 h at room temperature with 500 units benzonase (EMD Millipore, United States) and 0.5 mM MgCl_2_ (Sigma-Aldrich, United States); incubation for 2 h at 56 °C with 1 mg.mL^-1^ proteinase K (Interchim, France); incubation for 2 h at 37 °C with 0.25 mg.mL^-1^ RNase A (Qiagen, Germany). The extract was then sequentially treated with one volume of phenol (Interchim, France), centrifuged at 11,000 *g* for 10 min, and the aqueous phase recovered; one volume of phenol–dichloromethane (1:1, Interchim, France) with centrifugation and recovery of the aqueous phase; and one volume of dichloromethane, followed by centrifugation and collection of the aqueous phase. DNA was precipitated for 2 h at −20 °C with two volumes of absolute ethanol (VWR International, United States) and 0.15 M sodium acetate (Sigma-Aldrich, United States), centrifuged at 11,000 *g* for 30 min, and the pellet washed twice with ethanol/TE buffer pH 7.5 (7:3). DNA was resuspended in 200 µL of DNase-free water. For PCR screening of yeast clones, cultures were grown overnight at 30 °C in 50 µL SD-TRP selective medium with agitation, diluted with 100 µL water, and 5 µL of each suspension used as PCR template. Taq polymerase (Qiagen, Germany) was used with an initial denaturation step of 15 min at 98 °C (Bonnet et al., 2013). Primers are listed in Supplementary Table 2.

### 2.7 Plasmid extraction

For plasmid DNA extraction, transformed EPI300 cultures were grown overnight in 150 mL selective medium supplemented with CopyControl Induction Solution (LGC Biosearch Technologies, United States). Transformed yeast cultures were cultivated for 48 h in 150 mL selective medium. DNA extractions were performed using Plasmid DNA Midiprep kits (Qiagen, Germany) and concentrated with NucleoSnap Finisher Midi (Macherey-Nagel, Germany) following manufacturer instructions. Prior to extraction, yeast cells were treated for 1 h with 800 µL of a zymolyase 20 T stock solution (1% zymolyase 20T, 50 mM Tris-HCl, 25% glycerol; Seikagaku, Japan) and 80 µL of 14.3 M 2-mercaptoethanol (Sigma-Aldrich, United States). No specific modifications to these protocols were required due to the size of the constructs.

### 2.8 Transformation

EPI300 chemically competent cells were prepared by growing cultures at 37 °C in SOB medium to OD_600_ ≈ 0.6, washing, and freezing at −80 °C in CCMB solution (10 mM potassium acetate, 80 mM CaCl_2_, 20 mM MnCl_2_, 10 mM MgCl_2_, 10% glycerol in sterile water). TransforMax EPI300 (LGC Biosearch Technologies, United States) was used for transformation of yeast-derived DNA. Electroporation was performed with 50 µL competent cells and 2 µL yeast DNA extract in 1 mm cuvettes using a Bio-Rad Gene Pulser II (United States) at 2.5 kV, 200 Ω and 25 µF. Restriction profiling of pYCC3-VACV plasmids was performed by digesting 500 ng of *E. coli* extract with SalI (New England Biolabs, United States), followed by migration on a 0.8% agarose gel at 120 V for 1 h.

### 2.9 Restriction enzymes

Restriction enzymes used in this study were EcoRI, AfeI, PacI, SphI, and SalI (New England Biolabs, United States), SgrDI, MreI and FspAI (Thermo Fisher Scientific, United States).

### 2.10 Yeast spheroplast transformation

To prepare yeast spheroplasts competent for large DNA fragments, a fresh VL6-48 colony was inoculated into 20 mL YPDA and grown overnight, then used to inoculate 50 mL YPDA at an initial OD_660_ of 0.4 and cultured until OD_660_ reached 2–4. Cells were harvested by centrifugation at 1,000 *g* for 5 min, washed once with 30 mL sterile water and once with 20 mL 1 M D-sorbitol, then resuspended and incubated for 25 min in 20 mL SPE solution (1 M D-sorbitol, 10 mM Na_2_HPO_4_, 10 mM EDTA) containing 0.1% (v/v) of zymolyase 20 T stock solution (1% zymolyase 20T, 50 mM Tris-HCl, 25% glycerol; Seikagaku, Japan) and 0.2% 14.3 M β-mercaptoethanol (Sigma-Aldrich, United States). After centrifugation at 500 *g* for 10 min, the pellet was washed twice with 30 mL of 1 M D-sorbitol and resuspended in 1 mL STC solution (1 M D-sorbitol, 10 mM Tris-HCl, 10 mM CaCl_2_). For each transformation, 100 µL of spheroplast suspension was used (Kouprina and Larionov, 2008).

For construction of pYCC3-VACV, DNA was mixed as follows: 0.5 µg AfeI-digested pYCC3, 1 µg VACV DNA digested with SgrDI, 1 µg digested with MreI, and 1 µg digested with FspAI (restriction enzymes from New England Biolabs, United States, and Thermo Fisher Scientific, United States). For viral shuffling, 0.5 µg PacI-digested pYCC3-VACV was mixed with 3 µg CPXV DNA digested with SphI and 3 µg RPXV DNA digested with PacI. Spheroplasts and DNA were incubated together for 10 min at room temperature, followed by incubation for 10 min with 800 µL PEG8000 solution (20% PEG8000, 10 mM CaCl_2_, 10 mM Tris-HCl). After centrifugation at 500 *g* for 5 min, pellets were resuspended in SOS solution (1 M D-sorbitol, 0.65 mM CaCl_2_, 0.25% yeast extract, 0.5% peptone) and incubated for 40 min at 30 °C without agitation. The suspension was mixed with 7 mL of melted selective medium cooled to 50 °C and poured onto selective plates, which were incubated for 5 days at 30 °C. Small patches of primary transformants were then transferred to SD-TRP agar plates and incubated for 2 days at 30 °C.

### 2.11 Viral rescue

To restore the infectivity of viral DNA, 1 × 10^5^ A^54^9 or HeLa cells were infected in 24-well plates at a multiplicity of infection (MOI) of 1 using MVA diluted in 200 µL of phosphate-buffered saline (PBS) 1x (Sigma-Aldrich, United States) supplemented with 1% FBS, 0.6% magnesium acetate and 0.75% calcium chloride, and incubated for 1 h at 37 °C in 5% CO_2_ (Erbs et al., 2008). Transfection of 500 ng yeast-extracted DNA was performed using the Lipofectamine 3,000 kit (Thermo Fisher Scientific, United States) according to the manufacturer’s instructions. Plates were incubated for 7 days at 37 °C in 5% CO_2_, and 500 µL of supplemented DMEM was added 24 h after transfection. After 7 days, the 24-well plates were frozen at −20 °C for 24 h, and cells together with their supernatant were harvested and sonicated (Vibra-cell 72,408, Bioblock Scientific, France) for 30 s at 40% amplitude. A volume of 50 µL of each lysate was then used to infect 2 × 10^5^ A^54^9 or HeLa cells in 6-well plates, followed by incubation for 3 days at 37 °C in 5% CO_2_.

### 2.12 Viral DNA extraction from infected cells

For viral DNA sequencing, 2 × 10^5^ A^54^9 cells seeded in 6-well plates were infected with VACV or with an isolated viral clone in 250 µL PBS 1x supplemented with 1% FBS at 37 °C in 5% CO_2_ for 30 min. After infection, cells were overlaid with supplemented DMEM and incubated for 3 days at 37 °C in 5% CO_2_. Plates were then frozen at −20 °C, thawed slowly, and the resulting lysates were sonicated for 30 s at 40% amplitude. A volume of 100 µL of each infectious lysate was mixed with 5 mL supplemented PBS and used to infect an 80% confluent T175 flask of A549 cells for 30 min at 37 °C. Infected flasks were incubated for 3 days at 37 °C in 5% CO_2_ and subsequently frozen at −80 °C. Thawed cell suspensions were centrifuged for 3 min at 1,000 x g, the supernatant discarded, and the pellet resuspended in 9 mL PBS 1x. Viral DNA was extracted from 1 mL of this suspension using the Monarch HMW DNA Extraction Kit (New England Biolabs, United States) according to the manufacturer’s instructions.

### 2.13 Cytotoxicity assay

The lytic capacity of viruses was assessed using the CellTiter-Blue cell viability assay. HeLa, A549, HCT116 and MIA PaCa-2 cells were infected in suspension with serial virus dilutions corresponding to multiplicities of infection (MOI) ranging from 3 × 10^−5^ to 1, after which 1 × 10^4^ cells per well were seeded into 96-well plates in 150 µL of supplemented DMEM. Infected cells were incubated for 4 days at 37 °C. Cell viability was then quantified using the CellTiter-Blue assay (Promega, United States), and fluorescence readings were obtained using a Spark® multimode microplate reader (Tecan, Switzerland). All conditions were analyzed in triplicate, and non-infected cells were used as negative controls to establish the 100% survival reference for each assay.

### 2.14 Syncytia formation assay

HeLa cells were infected with the indicated viruses at a multiplicity of infection (MOI) of 10^–2^ or 10^–3^, and 3 × 10^5^ cells per well were seeded into 6-well culture dishes. Infected cells were incubated at 37 °C in a humidified 5% CO_2_ atmosphere and examined by optical microscopy 72 h post-infection to assess syncytia formation.

### 2.15 Comet assays

A549 cells were seeded into 60 mm tissue culture dishes and grown to confluence overnight. The following day, monolayers were washed with PBS and inoculated with approximately 40 plaque-forming units (PFU) of the indicated viruses in 500 µL PBS supplemented with 2% fetal bovine serum (FBS) and 1% of the following solution: 46.6 mM magnesium acetate and 68 mM calcium chloride (CaCl_2_), followed by incubation for 1 h at 37 °C. After adsorption, monolayers were rinsed and overlaid with 5 mL growth medium. Two days post-infection, the cell monolayers were fixed and stained with a 2.3% crystal violet solution (Sigma-Aldrich, United States) diluted 1:40 in absolute ethanol (VWR International, United States) to visualize viral plaques.

### 2.16 *In vitro* virus yield

To evaluate viral replication in human tumor cells, A549 cells were infected in 6-well plates at an MOI of 10^–3^ in triplicate. Three days post-infection, both supernatants and cell fractions were collected, subjected to one freeze thaw cycle, sonicated, and the viral progeny were quantified on Vero cells by plaque assay. To quantify infectious extracellular envelopped virus (EEV) and intracellular mature virus (IMV) at early time points, confluent A549 cells (1 × 10^6^ cells) were infected in 6-well plates at an MOI of 10^–1^ for 1 h in triplicate, washed with PBS, and overlaid with fresh medium before incubation for 24 h at 37 °C. Supernatants were collected to quantify EEV, whereas cell-associated virions (IMV) were recovered by resuspending the cell pellets in 1 mL PBS, followed by freeze thawing and sonication. Both EEV and IMV fractions were titrated on Vero cells by plaque assay. Plaque assays were performed on confluent Vero cell monolayers using standard procedures and enumerated after 3 days of incubation (Cotter et al., 2017).

### 2.17 DNA sequencing

Viral DNA samples extracted using NEB Monarch HMW DNA Extraction Kit were internally sequenced using a MinION™ (Oxford Nanopore Technologies®) to generate long read sequence data for analysis. Rapid sequencing kit (SQK-RBK114) and MinION/GridION Flow Cell were used following manufacturer’s instructions (Oxford Nanopore Technologies®) to prepare and sequence extracted DNA. The raw data files from R10.4.1 MinION flow cells were transferred from the lab to a high computation workstation. A data processing workflow, developed in-house to ensure reproducibility and automation of the analysis steps, has then been applied using a custom Snakemake pipeline. The steps are described hereafter. The raw electric signals (pod5 files) are converted into nucleic acid bases (fastq files) using guppy_basecaller software (v6.5.7) with dna_r10.4.1_e8.2_260bps_sup.cfg model file. The demultiplexing step was performed with guppy_barcoder (v6.5.7) to assign specific long reads to the respective sample. A quality control with pycoQC (v2.5.2) was used to generate metrics from the sequencing run including number of reads, total bases, N50, median read length, quality PHRED scores and barcode proportions. For each sample, reads are trimmed from the adapters with porechop (v0.2.4) and are filtered using NanoFilt (v2.8.0) with a minimum average PHRED quality score of Q12. Canu (v2.2) was then used for the *de novo* assembly from the filtered reads in combination with Medaka (v1.8.0) for the final polishing step. The contigs from each sample were imported in the Geneious software (v.8.1.9) for visualization and sequence alignments against the theoretical reference sequence. The filtered reads were then mapped with minimap2 on the longest contig of each sample to check the variations (single nucleotide variants, insertions, deletions) by visual inspection with IGV (v2.11.9).

### 2.18 Statistical analysis

Data were analyzed using one-way analysis of variance (ANOVA) followed by post-hoc comparisons with Tukey’s adjustment. Differences were considered statistically significant at p < 0.05, with significance levels indicated as follows: p < 0.05 (*) and p < 0.01 (**).

## 3 Results

### 3.1 TAR cloning strategy and virus rescue

The schematic overview of the construction of an infectious clone of VACV is presented in Figure 1. The VACV genome spans approximately 191 kb (Scott et al., 1990), comprising a unique central genomic region of approximately 165 kb that encodes around 247 putative open reading frames (ORFs). Flanking this region are the 5′and 3′inverted terminal repeats (ITRs), each comprising three distinct structural elements: i) coding ITRs (ITRc), approximately 8 kb in length, containing ∼13 conserved ORFs; ii) repeat regions (RRs), approximately 5 kb of short tandem repeats; and iii) covalently closed hairpin termini (Hp), approximately 0.15 kb in length, located at both ends of the genome and linking the complementary DNA strands into a continuous molecule (Baroudy et al., 1984). In *S. cerevisiae*, the presence of both 5′and 3′inverted terminal repeats (ITRs) within a single plasmid induces significant instability, most likely due to homologous recombination between the ITR sequences. This recombination event leads to the deletion of the intervening genomic region, resulting in a truncated plasmid that lacks the core of the viral genome. To circumvent this issue, the genome of the VACV Copenhagen strain was assembled into a yeast plasmid containing the core genome, the 5′ITRc and the 5′RR region, thereby improving plasmid stability.

**FIGURE 1:**
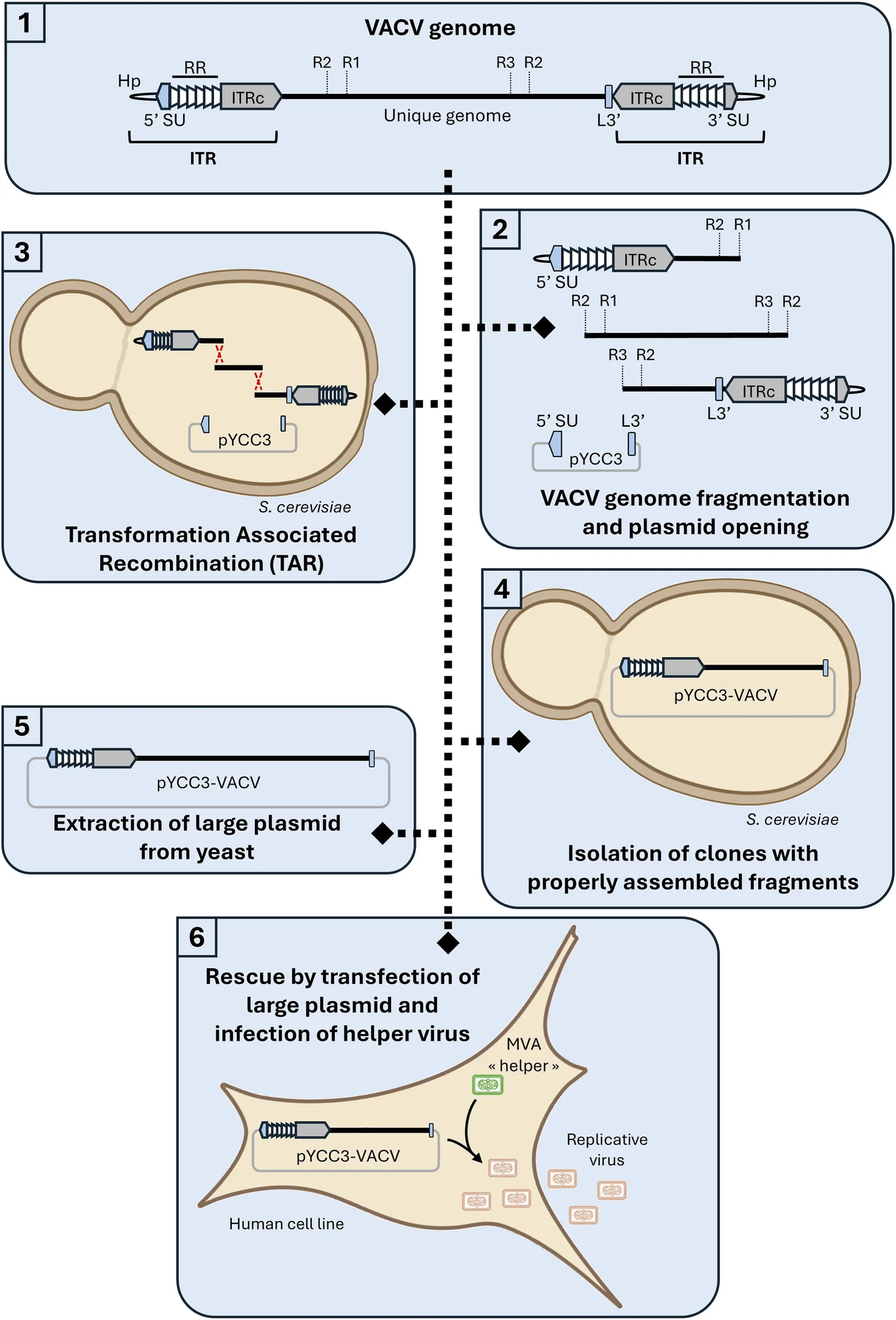
Strategy of the VACV genome assembly and virus rescue. Overlapping viral DNA fragments are assembled in yeast into a synthetic genome, which is subsequently reactivated in mammalian cells using a helper virus, enabling the generation of infectious viral particles.

Yeast centromeric and auxotrophic selection markers were introduced into an *E. coli* single-copy vector using conventional molecular biology techniques (Wittek and Moss, 1980). The vector, pYCC3, was engineered to include hook sequences corresponding to the 5′SU and L3' regions of the VACV genome (Figure 1). The 5′SU hook excludes the 5′Hp region, which consists of imperfectly paired DNA strands that are covalently closed at the terminus, that cannot be accommodated in a circular double-stranded plasmid. The L3' hook is positioned at the boundary of the 3′end of the unique genomic region, within the B19 R gene, thereby excluding the 3′ITRc, RR, and Hp regions from recombination. TAR technology exploits the high-efficiency homologous recombination machinery of yeast to assemble multiple overlapping DNA fragments. In this strategy, three overlapping VACV fragments, designated R1, R2, and R3, were co-transformed with the pYCC3 vector into *S. cerevisiae*. Following successful assembly, the recombinant plasmid was extracted and transfected into human cells pre-infected with a helper virus. Because poxvirus DNA alone is not infectious (Moss, 2013), the Modified Vaccinia virus Ankara (MVA) strain was used as a helper virus. MVA is replication-deficient in human cells but provides essential viral proteins and complements missing genomic regions, enabling the rescue and replication of the recombinant VACV genome (P. Erbs, personal communication).

### 3.2 VACV genome assembly via TAR cloning

The VACV genome was fragmented using three restriction enzyme digestions, generating ∼70 kb fragments with overlapping sequences to facilitate homologous recombination. These three VACV fragments, along with the linearized pYCC3 vector, were co-transformed into *S. cerevisiae* spheroplasts using the TAR method. Of the 188 TRP^+^ yeast clones screened, 18 tested positive for the presence of viral DNA in the first round of PCR targeting VACV genomic sequences. A second round of PCR, targeting each of the three VACV genome fragments individually, was performed on seven of these clones. All seven were confirmed to contain the complete set of fragments (Figure 2A). DNA extracts from two representative yeast clones, C1 and C2, which carried the expected ∼188 kb construct, were electroporated into *E. coli*. Although transformation efficiency is typically low for large constructs, several bacterial clones were successfully obtained. Plasmid DNA from these clones, designated pYCC3-VACV_C1 (noted C1) and pYCC3-VACV_C2 (noted C2), was validated by restriction enzyme profiling (Figure 2B). Subsequent analysis using Nanopore sequencing confirmed the integrity of the assembled VACV genome. The viral genome was precisely inserted between the 5′SU and L3' hook sequences within the pYCC3 vector, with no mutations detected across the assembled region. Notably, the 5′RR region was found to be shorter in both clones compared to the reference genome. However, this region is known to be hypervariable, and its truncation does not interfere with downstream applications or viral functionality (Wittek and Moss, 1980; Moss et al., 1981).

**FIGURE 2:**
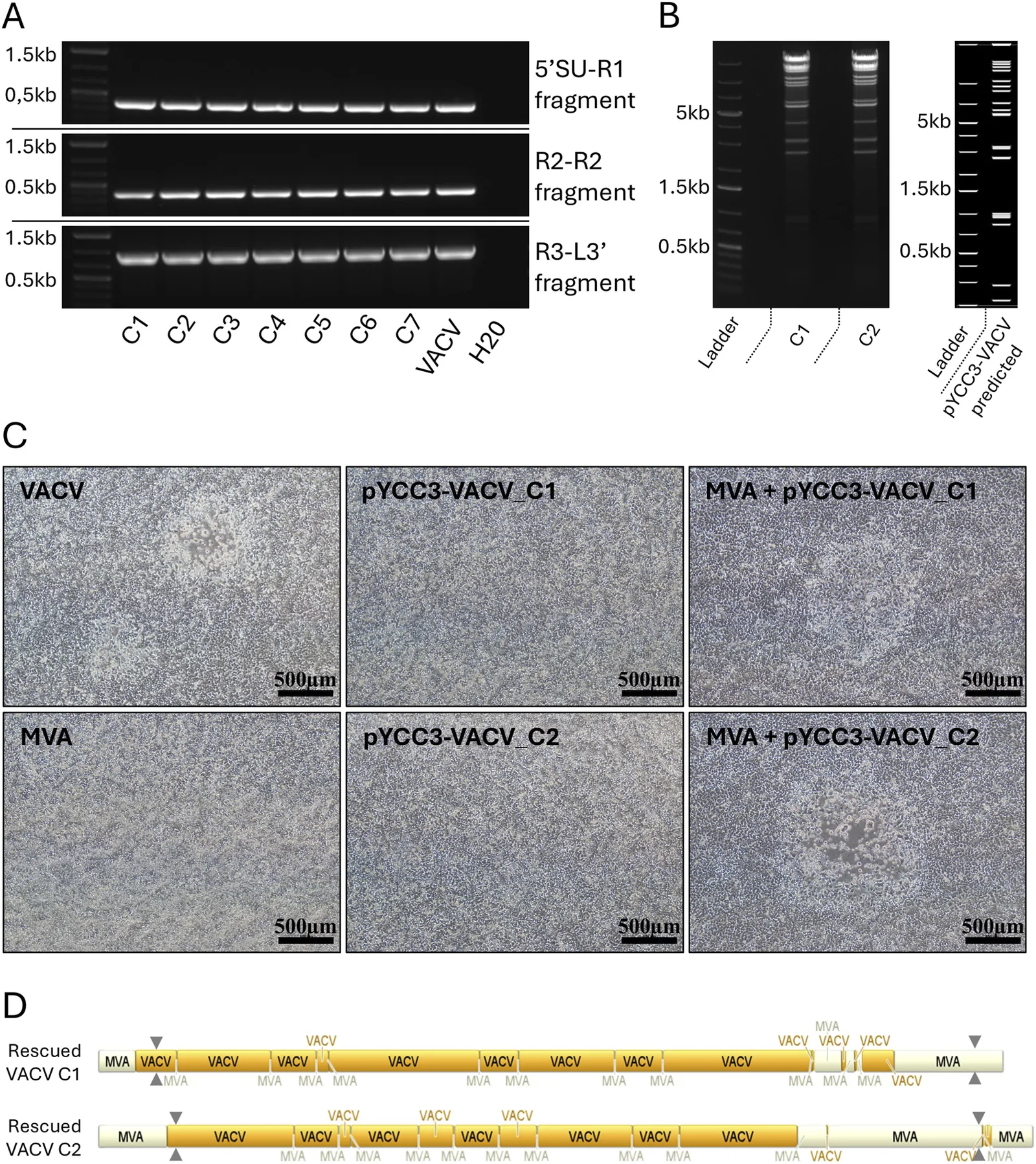
Analysis of isolated clones from TAR to rescue. **(A)** PCR analysis of the three VACV genomic fragments in selected TAR-derived yeast clones. **(B)** SalI restriction analysis of plasmid DNA purified from clones C1 and C2. **(C)** Plaque phenotype of rescue products obtained from pYCC3-VACV_C1 and pYCC3-VACV_C2 following reactivation in MVA-infected cells, compared with parental VACV and MVA. **(D)** Distribution of parental viral sequences within rescued clones derived from pYCC3-VACV_C1 and pYCC3-VACV_C2 based on Nanopore sequencing. Grey arrows indicate the identified boundaries of the terminal ITR regions. The complete terminal regions were not fully resolved by sequencing.

### 3.3 Rescue and characterization of synthetic infectious VACVs

To verify that the VACV genome, lacking the 3′ITRc, 3′RR, and Hp regions, inserted into the pYCC3 vector could be complemented to generate infectious viral particles, a rescue experiment was performed (Figure 2C). Human A549 cells were first infected with MVA, followed by transfection with either pYCC3-VACV_C1 or pYCC3-VACV_C2, both plasmids previously isolated from *S. cerevisiae*. No cytopathic effect was observed in cells infected with MVA alone or in cells transfected with the plasmids alone. In contrast, cell lysis was clearly observed in MVA-infected cells transfected with either pYCC3-VACV_C1 or pYCC3-VACV_C2, forming plaques similar to those induced by wild-type VACV. Independent rescue experiments were performed four times using pYCC3-VACV_C1 and four times using pYCC3-VACV_C2. Infectious viruses were recovered in all rescue attempts. One plaque from a rescued virus derived from pYCC3-VACV_C1 and one plaque from a rescued virus derived from pYCC3-VACV_C2 viruses were isolated and subjected to genome sequencing (Figure 2D). Sequencing confirmed the presence of VACV genomes derived from the pYCC3-VACV constructs. As expected, the missing genomic regions were complemented by sequences from the MVA genome. Recombination between VACV and MVA occurred differently in the two clones, although both exhibited similar overall genomic profiles. In the 5′region, recombination restored the Hp structure using MVA-derived sequences. In the 3′region, the 3′ITRc, RR, and Hp were also complemented by MVA. Additionally, small MVA-derived insertions were detected throughout the rescued VACV genomes. These results demonstrate that the pYCC3-VACV plasmid can be used for regenerating infectious VACV particles when complemented by MVA *in trans*.

### 3.4 TAR shuffling strategy

The schematic overview for constructing infectious chimeric poxviruses using TAR is presented in Figure 3. To generate shuffled chimeric genomes, the pYCC3-VACV plasmid, along with genomic DNA from Cowpox virus (CPXV) and Rabbitpox virus (RPXV), was co-transformed into *S. cerevisiae*. To enhance transformation efficiency and promote homologous recombination, the viral genomes were digested with restriction enzymes to produce fragments ranging from 10 to 40 kb. The restriction enzyme used for pYCC3-VACV was specifically selected to avoid cleavage within the vector backbone, thereby preserving its structural integrity during recombination. Two TRP^+^ clones were obtained when *S. cerevisiae* was transformed with digested pYCC3-VACV alone and none with digested CPXV and RPXV genomic DNA alone. In contrast, 42 TRP^+^ clones were recovered when *S. cerevisiae* was co-transformed with digested pYCC3-VACV and digested genomic DNA from CPXV and RPXV, indicating successful recombination events. To evaluate the formation of chimeric viral DNA constructs, plasmids were extracted from ten TRP^+^ yeast clones and subjected to a rescue assay using MVA as a helper virus in HeLa cells. As previously described, no cytopathic effect was observed in cells infected with MVA alone or transfected with plasmids alone. However, in MVA-infected cells transfected with plasmids isolated from *S. cerevisiae*, five clones successfully produced infectious viral particles, confirming the feasibility of TAR-based shuffling for synthetic poxvirus engineering.

**FIGURE 3:**
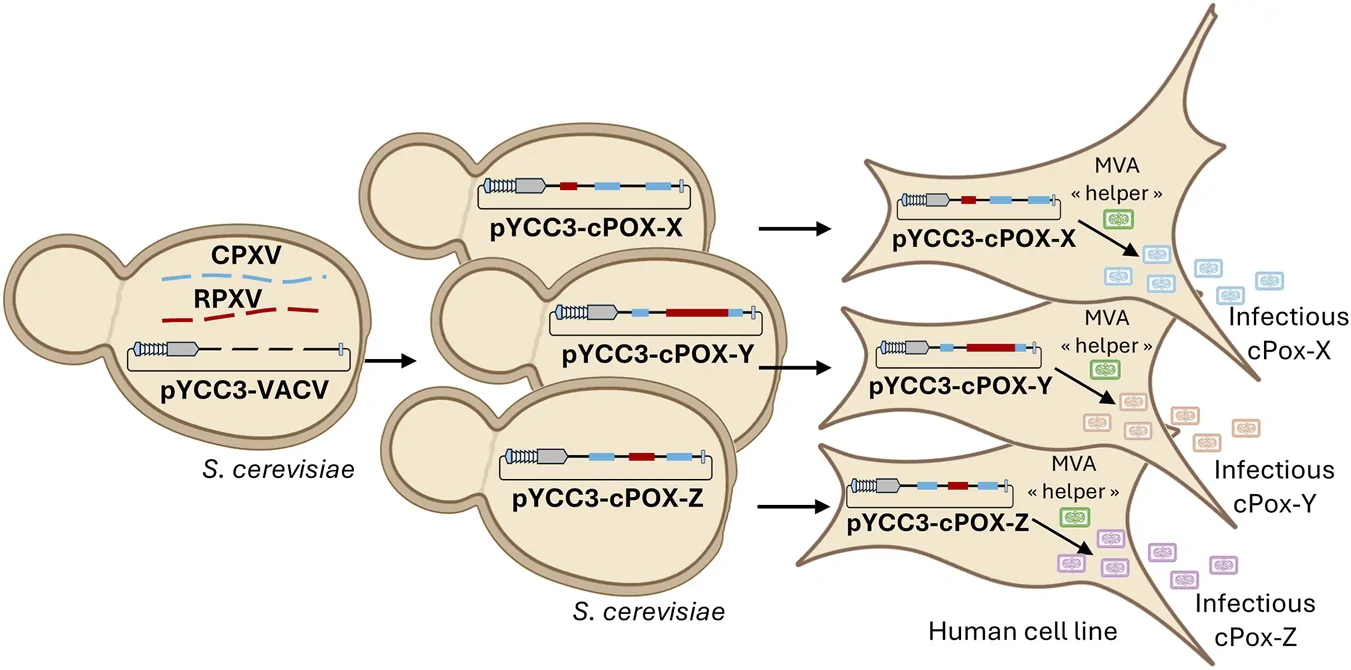
TAR-based shuffling of CPXV and RPXV genomes using the pYCC3-VACV plasmid. Fragmented pYCC3-VACV plasmid DNA and genomic DNA from CPXV and RPXV are co-transformed into yeast cells. Homologous recombination generates chimeric plasmids, which are then extracted and transfected into mammalian cells pre-infected with MVA to produce infectious viruses.

### 3.5 Characterization of synthetic infectious chimeric poxviruses

Plaque morphology and syncytia formation were evaluated in HeLa cells (Figure 4A; Supplementary Figure S1A), and viral dissemination was assessed by comet assay in A549 cells (Figure 4B; Supplementary Figure S1B) for the parental viruses and the cPOX variants. Among the parental strains, VACV and CPXV produced regular plaques without syncytia or comet formation, whereas RPXV generated syncytial plaques in HeLa cells and exhibited a spreading phenotype in A549 cells. The cPOX01-02 clone displayed VACV-like lytic plaques in both HeLa and A549 cells but showed limited dissemination. cPOX02-03 formed syncytial plaques with irregular contours in HeLa cells and demonstrated low spreading in A549 cells. cPOX04-12 produced small RPXV-like plaques in HeLa cells and extensive comet formation in A549 cells. cPOX05-14 exhibited irregular lysis with small syncytia in HeLa cells and lacked comet formation in A549 cells. Finally, cPOX06-18 generated small, dispersed plaques with syncytia in both HeLa and A549 cells.

**FIGURE 4:**
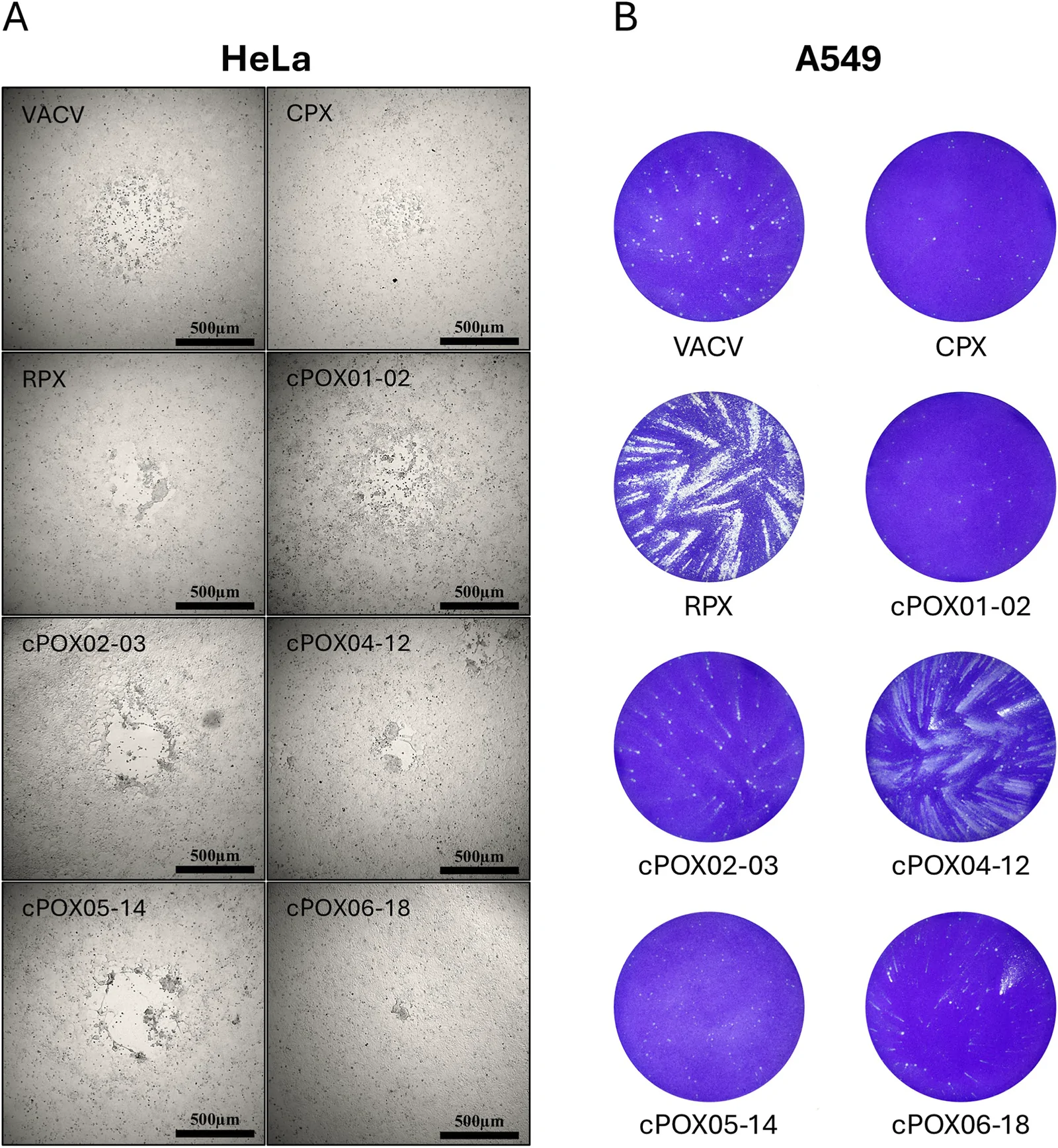
Plaque morphology and viral spread of chimeric viruses. **(A)** Brightfield microscopy images of plaque assays performed on HeLa cells infected with parental viruses (VACV, CPXV, RPXV) and with chimeric viruses rescued from TAR-shuffling clones (cPOX01-02, cPOX02-03, cPOX04-12, cPOX05-14, and cPOX06-18) **(B)** Representative comet-shaped plaques observed in A549 cells infected with the indicated viruses, illustrating differences in viral spread and extracellular envelopped virus (EEV) production. A549 monolayers were infected with the indicated viruses, incubated for 48 h, and stained with crystal violet to visualize comet formation, reflecting enhanced viral spread.

Oncolytic potency was evaluated across four human tumor cell lines (A549, HeLa, HCT116, and MIA PaCa-2) by determining half-maximal effective concentrations (EC_50_) values following infection with serial dilutions of each virus (Figure 5). Among the parental strains, VACV exhibited strong activity with EC_50_ values ranging from 1.2 × 10^−2^ to 2.7 × 10^−2^ MOI, whereas CPXV and the non-propagative MVA were markedly less potent (EC_50_ > 0.3 MOI). RPXV showed intermediate activity, particularly in HeLa cells (4.7 × 10^−2^ MOI). Notably, several cPOX variants demonstrated improved potency compared to VACV, with cPOX01-02 and cPOX04-12 achieving the lowest EC_50_ values in A549 (9 × 10^−3^ MOI) and HCT116 (1.9 × 10^−3^ MOI), respectively. These findings indicate that specific modifications in cPOX variants significantly improve oncolytic activity, particularly in colorectal and lung cancer cell lines.

**FIGURE 5:**
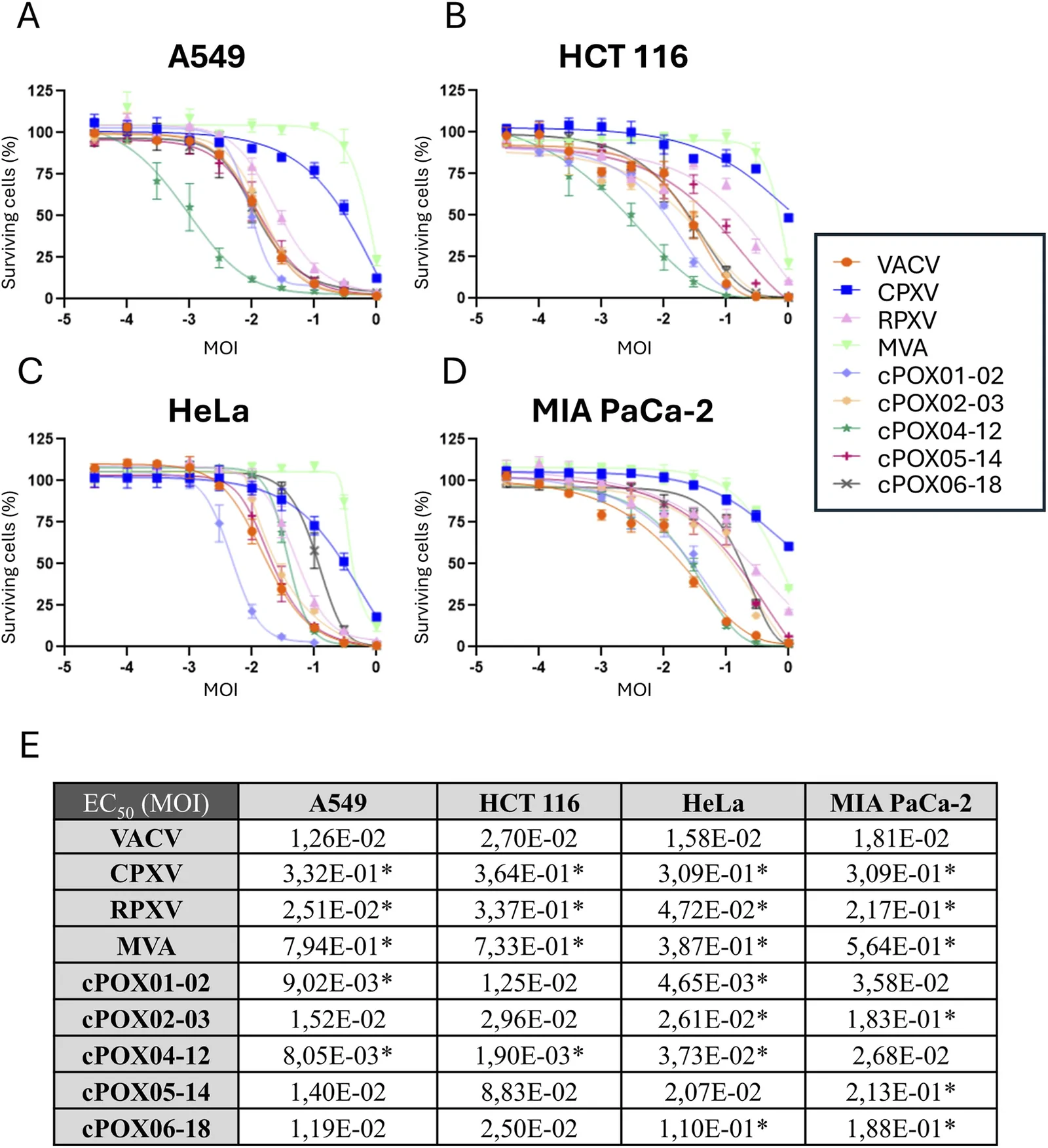
Oncolytic activity across a panel of human tumor cell lines. **(A–D)** A549 **(A)** HCT 116 **(B)** HeLa **(C)** and MIA PaCa-2 **(D)** cells were infected with serial dilutions of each virus. Cell viability was assessed 4 days post-infection using the CellTiter-Blue assay and normalized to uninfected controls. Data represents the mean ± SD from three independent experiments. **(E)** EC_50_ values for each virus in each cell line were calculated by fitting a sigmoidal dose–response curve to the viability data shown in panels A–D. Statistical significance was determined relative to the VACV group using one-way ANOVA: p < 0.05 (*).

All tested viruses replicated in A549 cells (Figure 6A), with VACV exhibiting the highest amplification, reaching approximately 2 × 10^5^ fold. CPXV and RPXV exhibited intermediate amplification (∼10^5^-fold), while cPOX04-12 reached levels comparable to VACV. Other chimeric strains displayed variable replication, with cPOX01-02 being the least efficient (∼10^4^ fold). Analysis of EEV production revealed marked differences among strains (Figure 6B). VACV and CPXV produced minimal EEV (<0.2%), RPXV reached ∼4%, and cPOX04-12 showed the highest proportion (∼9%). These EEV percentages are consistent with comet formation observed, with cPOX04-12 exhibiting extensive spreading. Together, these data indicate that cPOX04-12 combines strong replication capacity with enhanced EEV release, features that may favor efficient cell-to-cell spread.

**FIGURE 6:**
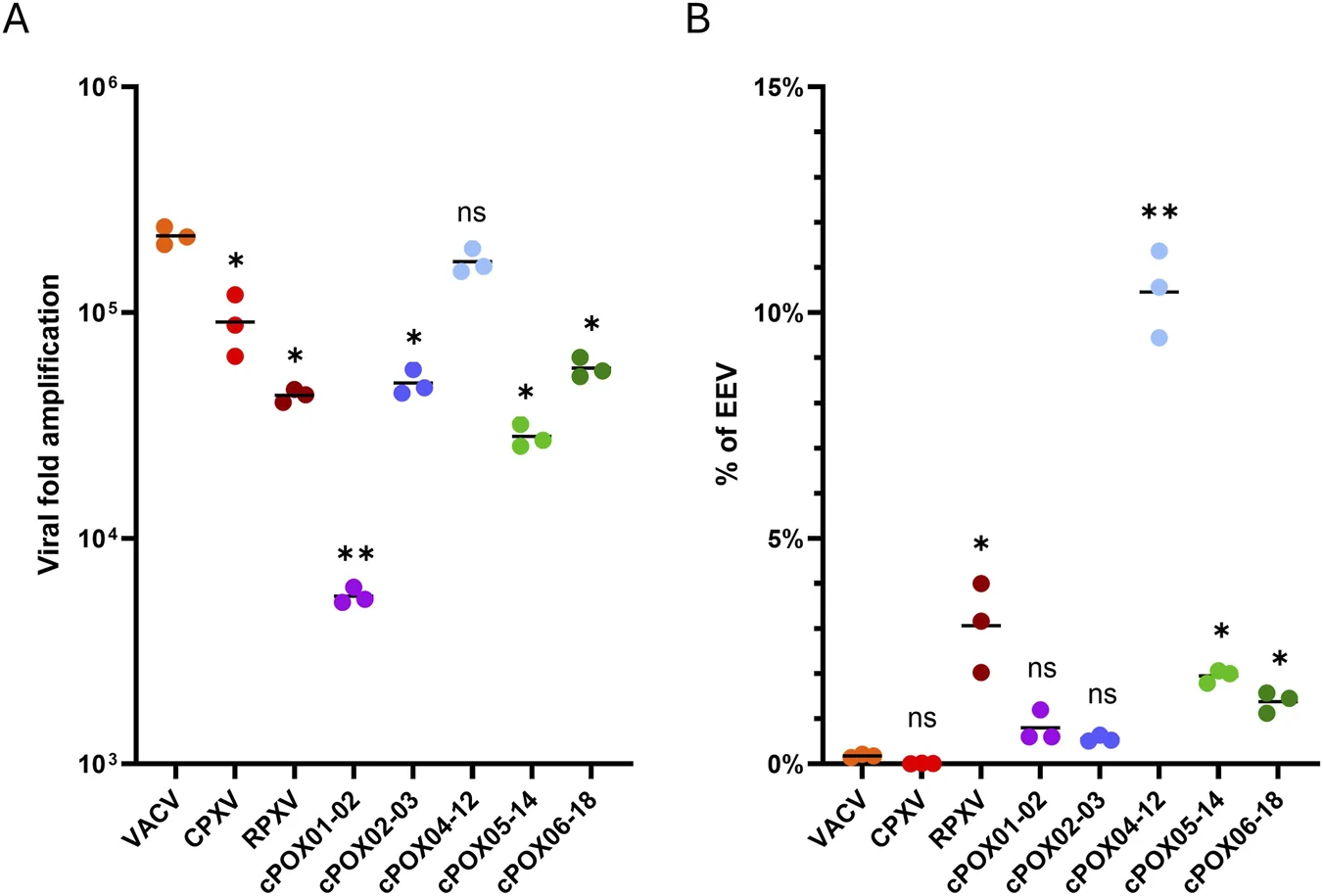
Replication and early production of EEV in tumor cells. **(A)** Viral amplification in A549 cells. Monolayers were infected at a MOI of 10^–3^. At 3 days post-infection, both supernatants and cells were collected, and viral progeny were quantified by plaque assay. Results are expressed as fold amplification, calculated as the output/input ratio. Data represents results from three independent experiments. The horizontal line represents mean. Statistical significance was assessed against the VACV group using one-way ANOVA: not significant (ns), p < 0.05 (*), p < 0.01 (**). **(B)** Ratio of EEV to total progeny virus (IMV + EEV). A549 cell monolayers were infected with the indicated viruses at a MOI of 0.1. At 1 day post-infection, viral titers were determined for EEV (supernatant only) and total progeny virus (IMV + EEV; combined supernatants and cells). Data represents results from three independent experiments. The horizontal line represents mean. Statistical significance was assessed against the VACV group using one-way ANOVA: not significant (ns), p < 0.05 (*), p < 0.01 (**).

### 3.6 Sequence analysis

To identify recombination events that occurred during the TAR cloning and rescue process, the five clones were subjected to whole-genome sequencing using nanopore sequencing technology (Figure 7). For each clone, unique recombination events involving sequences from VACV, CPXV, RPXV, and MVA were identified. Sequenced genome sizes for the different clones ranged from 150 kb to 187 kb. However, the inverted terminal repeats (ITR) were only partially covered by sequencing, preventing any conclusions regarding their structure, function, or potential variability. The presence of CPXV and RPXV genomic segments confirms that homologous recombination occurred in *S. cerevisiae* during co-transformation with the pYCC3-VACV vector. Additionally, sequences derived from the MVA genome were found in the rescued viruses, indicating that recombination between the chimeric plasmid and MVA took place during the rescue step in human cells. These findings demonstrate that infectious chimeric viruses can be generated from four distinct parental genomes, highlighting the versatility of the TAR shuffling strategy for synthetic virology and oncolytic virus development. Moreover, the observed phenotypic diversity and variable oncolytic potential among the clones underscore the utility of this approach for engineering novel viral therapeutics.

**FIGURE 7:**
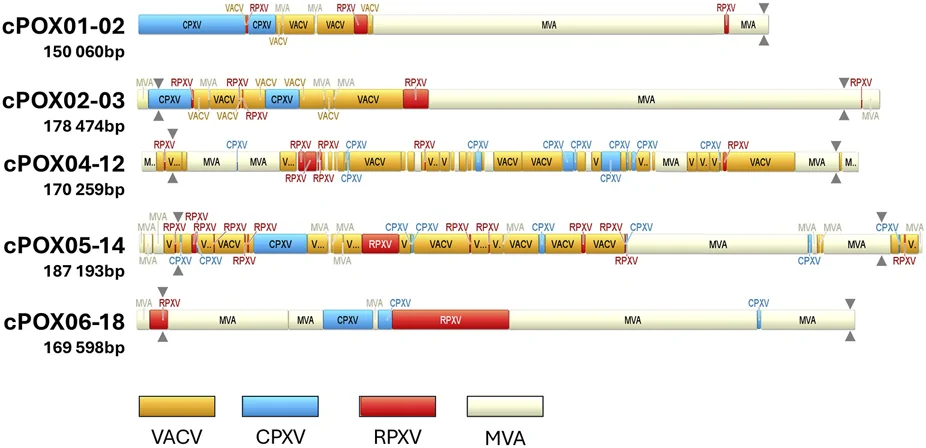
Genomic map of cPOX viruses showing the origin of genomic segments. Genomic regions are color-coded according to their respective parental viruses. Each annotation highlights the longest contiguous segment displaying the highest sequence identity with the corresponding parental genome. Genome sizes are indicated beneath each virus name. Grey arrows indicate the identified boundaries of the terminal ITR regions. The complete terminal regions were not fully resolved by sequencing. The 5′ITR boundary of clone cPOX02-01 could not be identified.

## 4 Discussion

In this study, we establish a yeast-based genome shuffling platform that enables the generation of infectious chimeric poxviruses from multiple parental species, thereby expanding the accessible sequence–function landscape for oncolytic virus engineering.

Chimeric virus generation represents a powerful strategy for engineering novel OVs with enhanced therapeutic properties (Kuhn et al., 2008; El-Andaloussi et al., 2012; O’Leary et al., 2018; Ricordel et al., 2018a). By combining genetic elements from different viral backbones, this approach enables the rational design of recombinant viruses that integrate desirable features such as improved tumor selectivity, enhanced immunogenicity, and optimized replication kinetics. Such chimeric constructs can overcome limitations inherent to individual parental strains and offer a flexible platform for tailoring OVs to specific clinical contexts.

TAR cloning has been widely applied to the reconstruction of both DNA virus genomes and infectious DNA copies of RNA viruses, highlighting its versatility in viral synthetic genomics (Larionov et al., 1996; Gibson et al., 2008; Oldfield et al., 2017; Labroussaa et al., 2021). It has enabled the successful cloning of viral genomes across a wide size range, from the 10 kb of potyviruses to the 235 kb of human cytomegalovirus (Desbiez et al., 2012; Brown et al., 2017; Thi Nhu Thao et al., 2020). Additionally, TAR has facilitated the assembly of large DNA viruses such as ascoviruses and baculoviruses, demonstrating its broad applicability in synthetic virology (Shang et al., 2017; Zaghloul et al., 2025).

Despite recent advances in yeast-based assembly of large viral genomes, poxvirus genomes have remained particularly challenging to manipulate because of their large size and structural complexity, including covalently closed hairpin termini and extensive ITRs. Notably, Chen et al. recently used TAR to clone and engineer a replication-defective mpox virus genome in yeast, demonstrating the feasibility of yeast-based manipulation of orthopoxvirus genomes (Chen et al., 2025). However, the generation of infectious chimeric orthopoxviruses through genome shuffling in yeast had not been reported.

In this study, we extend this approach by applying TAR to assemble a near-complete VACV genome and generate chimeric oncolytic poxviruses. Our results demonstrate that TAR-mediated genome shuffling can be successfully applied to orthopoxvirus genomes, enabling the generation of infectious chimeric viruses through homologous recombination in yeast. These findings expand the scope of TAR cloning beyond genome capture and engineering, establishing a versatile platform for the generation and evolution of recombinant poxviruses and opening new avenues for yeast-based poxvirus engineering.

We first used TAR to isolate and assemble most of the vaccinia virus (VACV) genome, deliberately excluding the unstable 5′terminal hairpin and the complete 3′inverted terminal repeat (ITR) region. This approach enabled the reconstruction of 175 kb of the 191 kb VACV genome. To complement the missing essential terminal sequences, previously described rescue approaches using Fowlpox virus (FPV) or Shope Fibroma virus (SFV) were considered. However, these viruses failed to support recombination with VACV in cell lines, likely due to the genetic divergence between avipoxvirus, leporipoxvirus, and orthopoxvirus (Scheiflinger et al., 1992; Yao and Evans, 2003; Qin and Evans, 2014). To overcome this limitation, we performed virus reactivation using MVA, an attenuated orthopoxvirus that, despite being replication-deficient in human cells, shares sufficient genomic homology with VACV to enable recombination and successful rescue. Following reactivation with MVA, the synthetic VACV particles exhibited the expected genomic architecture, with MVA-derived sequences incorporated at both the 5′and 3′termini. This outcome was consistent with homologous recombination mechanisms occurring within cytoplasmic viral factories, where the synthetic VACV genome interacts with the MVA helper virus. The incorporation of MVA terminal sequences underscores the importance of selecting a helper virus with adequate genomic compatibility to ensure efficient rescue and structural integrity of the recombinant virus. Importantly, the synthetic VACV constructs generated through this approach were fully replication-competent in human tumor cell lines and displayed phenotypic characteristics indistinguishable from wild-type VACV, including replication kinetics, cytopathic effects, and plaque morphology. These findings confirm that the TAR-based assembly strategy, despite omitting terminal regions, preserves the essential functional integrity of the virus when complemented appropriately.

A second TAR-based design–build cycle was performed to integrate additional poxvirus genomes into the previously constructed plasmid containing the near-complete VACV genome. Fragmented genomes from CPXV and RPXV were co-transformed with pYCC3-VACV into *S. cerevisiae*, enabling *in vivo* homologous recombination and the generation of novel chimeric genomes. Due to the technical challenge of sequencing dozens of 180 kb single-copy plasmids directly in *S. cerevisiae*, ten clones were selected for direct rescue. Remarkably, half of these yeast clones yielded infectious viruses upon reactivation. Sequencing of the rescued viruses revealed the presence of sequences derived from VACV, CPXV, and RPXV, confirming that homologous recombination occurred in *S. cerevisiae* and successfully generated chimeric viral genomes. These results demonstrate that TAR cloning can efficiently produce random chimeric constructs, offering a high degree of genomic diversity.

Recombination in this system is driven by sequence homology between related orthopoxvirus genomes and is therefore expected to preserve an overall genomic organization similar to that of the parental viruses. Furthermore, the recovery of infectious viruses remains constrained by the biological requirements for poxvirus viability, as illustrated by the limited proportion of rescued infectious clones. Nevertheless, the development of any candidate derived from this platform will require evaluation of replication in non-transformed cells, genetic stability, tumor selectivity, and safety before advanced preclinical studies.

Non-infectious clones may result from incomplete genome assembly during TAR cloning, loss of essential viral sequences required for virion formation and replication, or the generation of non-viable chimeric genome configurations. Future large-scale sequencing of both infectious and non-infectious clones will help identify the genomic determinants associated with successful virus rescue.

A methodological complexity inherent to our approach is the use of MVA as a helper virus for genome rescue, which introduces an additional layer of recombination during reactivation in mammalian cells. This secondary recombination event can partially obscure the precise architecture and junctions of recombination events that initially occurred in *S. cerevisiae*. Therefore, the final genomes recovered after rescue represent the cumulative outcome of yeast-mediated genome shuffling and helper-virus–mediated complementation.

The presence of genomic segments derived from Cowpox virus (CPXV) and Rabbitpox virus (RPXV) within rescued viruses constitutes unambiguous evidence that recombination between these genomes occurred prior to rescue. Because CPXV and RPXV genomic DNA were introduced during the yeast recombination step and are absent from both the VACV backbone and the MVA helper virus, their integration into infectious chimeras is consistent with recombination events originating in yeast.

The comparative analysis of chimeric clones revealed substantial heterogeneity in lysis morphology, viral replication and oncolytic activity across various human cancer cell lines. While clones such as cPOX02-03 exhibited lysis patterns distinct from those of all parental viruses and were characterized by extensive syncytium formation, cPOX05-14 and cPOX06-18 displayed atypical lysis phenotypes associated with reduced activity. Notably, cPOX01-02 and cPOX04-12 demonstrated distinct functional profiles: cPOX01-02 outperformed VACV in HeLa cells despite displaying a VACV-like phenotype, while cPOX04-12 outperformed VACV in A549 and HCT 116 cells and additionally exhibited pronounced comet-like spreading. These findings underscore the functional diversity resulting from recombination-driven genome mosaicism and highlight the need for individual clone characterization to identify candidates with optimal therapeutic potential.

The 4 cell lines used in oncolytic activity assays shown in Figure 5 differ in their innate immune competence. A549 cells retain robust type I interferon signaling, whereas MIA PaCa-2 cells generally display weaker interferon responsiveness, with HCT116 and HeLa exhibiting intermediate phenotypes (Guo et al., 2022; Ji et al., 2023). Nevertheless, poxviruses encode multiple antagonists of antiviral innate immune responses, suggesting that the differences in oncolytic activity observed across cell lines are unlikely to be explained solely by interferon signaling and may also reflect virus-specific genetic determinants.

Plaque and comet assays revealed notable phenotypic differences between the isolated chimeric clones and their parental viruses. In HeLa cells, infection resulted in distinct lysis patterns, with cPOX02-03 inducing large syncytia, whereas clones cPOX05-14 and cPOX06-18 produced smaller syncytia.

The vaccinia virus genes A56 R and K2L are well-established regulators of virus-induced cell–cell fusion. A56 R encodes the viral hemagglutinin (Ichihashi and Dales, 1971; de Haven et al., 2011), while K2L encodes a serine protease inhibitor–like protein (Law and Smith, 1992); together, these proteins form a complex at the surface of infected cells that inhibits the viral fusion machinery and prevents syncytium formation. Consistent with this, mutation or deletion of either A56 R or K2L has been shown to result in extensive cell–cell fusion and the formation of multinucleated syncytia.

In the present study, all sequenced clones retained intact A56 R alleles derived either from MVA or VACV. Therefore, the observed syncytial phenotypes cannot be attributed to alterations in A56 R.

In the case of K2L, both cPOX01-02 and cPOX04-12 carry the CPXV-derived allele, despite exhibiting distinct phenotypes with respect to syncytium formation. Whereas cPOX01-02 did not induce syncytia, cPOX04-12 displayed a pronounced syncytial phenotype. The CPXV K2L allele has previously been described as an inhibitor of syncytium formation (Turner and Moyer, 1995). Consequently, the syncytia observed in cPOX04-12 are likely driven by additional genetic determinants that overcome or counteract the inhibitory effect of the CPXV-derived K2L protein.

In the other chimeric viruses, K2L sequences originated from parental viruses for which the role of K2L in the regulation of syncytium formation has not been characterized. Therefore, the contribution of these alleles to the observed phenotypes remains unclear.

Comet formation, a distinct cytopathic effect observed in poxvirus-infected monolayers, is closely associated with the release of EEV (Smith and Law, 2004). EEV particles are enveloped in a double membrane derived from the host cell and are released prior to cell lysis, enabling long-range dissemination of the virus. This phenotype is particularly relevant in the context of viral spread and immune evasion (Vanderplasschen et al., 1998). Comet formation was minimal in all chimeric clones except cPOX04-12, which displayed prominent comet-like structures in A549 cells, associated with a markedly higher proportion of EEV, showing an EEV production rate approximately 50-fold greater than that of VACV. The A34 R gene is the only poxvirus gene currently known to regulate EEV formation, with a single mutation at residue K151 (K151 E) shown to enhance EEV production up to 40-fold in the Vaccinia strain (Mcintosh and Smith, 1996). However, all sequenced chimeric clones, including cPOX04-12, carry the A34 R gene from MVA, which lacks the K151 mutation. These findings suggest that additional genetic determinants beyond A34 R may contribute to EEV production and comet formation in chimeric viruses, and their identification warrants further investigation. These findings underscore the potential of yeast-based TAR cloning as a powerful platform for generating diverse chimeric poxviruses. By enabling the shuffling of genomes from multiple parental strains, this approach provides access to a rich source of genetic and phenotypic diversity. Future studies leveraging high-throughput sequencing and phenotypic screening of larger chimeric virus libraries could facilitate genetic mapping of key traits such as syncytia formation, EEV production, oncolytic potency and immunogenicity. Indeed, identifying novel genetic determinants of EEV production and syncytium formation could contribute to the development of improved oncolytic viruses. Conversely, implementing the A56R and A34R gene mutations in our chimeric viruses could lead to a significant improvement in their oncolytic properties.

Moreover, expanding the platform to include poxviruses from other genera could further enhance the diversity and utility of engineered viruses. Ultimately, this strategy holds promise for the rational design of optimized oncolytic viruses capable of efficient tumor spread and immune evasion. A major advantage of our yeast-based synthetic workflow is the complete avoidance of *E. coli*. Cloning large and structurally complex viral genomes in *E. coli* using bacterial artificial chromosomes (BACs) presents well-documented technical challenges. For example, several groups have reported difficulties in cloning and manipulating coronavirus genomes due to their size and complexity, while high G + C content has been implicated in the cloning inefficiency of herpesviruses (Hall et al., 2012; Cao et al., 2023; Xiao et al., 2023; Knickmann et al., 2024). Instability of full-length viral cDNA clones in *E. coli* has been frequently observed for flaviviruses, herpesviruses, and coronaviruses, often due to toxicity induced by viral sequences during bacterial propagation (Polo et al., 1997; Almazán et al., 2000; Kanda et al., 2011; Yakushko et al., 2011). In some cases, cryptic bacterial promoters within viral genomes drive the expression of toxic proteins, as seen with Zika virus, where high-copy-number plasmids exacerbate this effect (Li et al., 2011). To overcome these limitations, yeast-based synthetic genomics approaches have emerged as attractive alternatives for the assembly and modifications of viral genomes (Rey et al., 2025). Our yeast-based synthetic approach enables the assembly of complex chimeric viral genomes without bacterial propagation. This strategy represents a robust and scalable alternative for the generation of new oncolytic poxviruses, facilitating rapid and precise genome engineering. Historically, Domi and Moss demonstrated the feasibility of cloning the VACV genome into a BAC, allowing stable maintenance and precise genetic manipulation of the full-length viral genome in *E. coli* (Domi and Moss, 2002). Their method relied on the accumulation of head-to-tail concatemers of VACV DNA, which were circularized and introduced into bacteria. This BAC-based system enabled efficient genome editing via recombineering and recovery of infectious virus through transfection into mammalian cells co-infected with a helper fowlpox virus (FPV). While well-suited for functional studies and complex genome engineering, this strategy requires careful control of concatemer formation and circularization, and the retention of bacterial sequences in the rescued virus may necessitate additional removal steps for translational applications. To overcome these limitations, Chiuppesi et al. developed a synthetic MVA platform that assembles the MVA genome from three synthetic BAC-cloned fragments and reconstitutes the virus via homologous recombination in permissive cells (Chiuppesi et al., 2020). This method enables rapid generation of recombinant viruses without plaque purification, and the spontaneous loss of bacterial sequences during virus rescue enhances its translational potential. Nevertheless, the system still relies on *E. coli* for BAC propagation, which may introduce bacterial elements or recombination artifacts. More recently, Gao et al. reported the complete synthetic assembly of the 180-kb MVA genome using 37 chemically synthesized DNA fragments and TAR technology (Gao et al., 2025). The resulting system, composed of five plasmids, enables rapid and modular construction of recombinant MVA vectors, significantly accelerating vaccine development. Despite its advantages, the method remains technically demanding and resource-intensive, and its reliance on *E. coli* for BAC propagation introduces potential concerns regarding plasmid stability and recombination fidelity.

Future investigations should focus on optimizing TAR cloning and virus rescue procedures to improve the recovery rate of infectious viruses and assess the reproducibility of the platform. Alternative strategies for the delivery of large viral genomes, as well as pooled rescue approaches, could further increase screening throughput. For candidates selected for further development, comprehensive genetic stability studies will be required. Another important area of research is the identification of the genetic determinants underlying syncytium formation and EEV release, which could be exploited to enhance intratumoral spread and modulate antitumor immune responses. In addition, studies evaluating biodistribution, viral shedding, and environmental safety will be essential. Finally, the impact of combining deletions or modifications of the viral genes coding for thymidine kinase (TK) and viral ribonucleotide reductase (RR) should be investigated, as this strategy may further enhance tumor selectivity by increasing viral dependence on the elevated nucleotide pools characteristic of proliferating cancer cells (Foloppe et al., 2019; Gallardo et al., 2020).

From a broader therapeutic perspective, these synthetic chimeric viruses hold potential for integration into combination regimens with immune checkpoint inhibitors, chemotherapeutics, or targeted agents (Yuan et al., 2024). Their modular design also permits the incorporation of transgenes encoding immunostimulatory molecules, further expanding their utility as multifunctional oncolytic platforms. Collectively, the strategy described herein lays the foundation for a new generation of customizable oncolytic viruses with improved efficacy and adaptability across diverse cancer types.

## Data Availability

The raw data supporting the conclusions of this article will be made available by the authors, without undue reservation.
